# Inflammatory Biomarkers as Predictors of Response to Immunotherapy in Urological Tumors

**DOI:** 10.1155/2019/7317964

**Published:** 2019-09-19

**Authors:** Giuseppe Schepisi, Nicole Brighi, Maria Concetta Cursano, Giorgia Gurioli, Giorgia Ravaglia, Amelia Altavilla, Salvatore Luca Burgio, Sara Testoni, Cecilia Menna, Alberto Farolfi, Chiara Casadei, Giuseppe Tonini, Daniele Santini, Ugo De Giorgi

**Affiliations:** ^1^Department of Medical Oncology, Istituto Scientifico Romagnolo per lo Studio e la Cura dei Tumori (IRST) IRCCS, Meldola, Italy; ^2^Medical Oncology Department, Campus Bio-Medico University, Rome, Italy; ^3^Biosciences Laboratory, Istituto Scientifico Romagnolo per lo Studio e la Cura dei Tumori (IRST) IRCCS, Meldola, Italy; ^4^Unit of Biostatistics and Clinical Trials, Istituto Scientifico Romagnolo per lo Studio e la Cura dei Tumori (IRST) IRCCS, Meldola, Italy

## Abstract

Immunotherapy represents the new era of cancer treatment because of its promising results in various cancer types. In urological tumors, the use of the immune-checkpoint inhibitors (ICIs) is increasingly spreading. Although not all patients and not all diseases respond equally well to immunotherapy, there is an increasing need to find predictive markers of response to ICIs. Patient- and tumor-related factors may be involved in primary and secondary resistance to immunotherapy: tumor-derived protein and cytokines, tumor mutational burden, and patient performance status and comorbidities can condition tumor response to ICIs. Recently, some of these factors have been evaluated as potential biomarkers of response, with conflicting results. To date, the expression of programmed death-ligand 1 (PD-L1) and the presence of deficient mismatch repair (dMMR) in tumor tissue are the only biomarkers capable of guiding the clinician's decision in urothelial cancer and prostate cancer, respectively. In this review, we performed a comprehensive search of the main publications on biomarkers that are predictive of response to ICIs in urological cancers. Our aim was to understand whether existing data have the potential to drive clinical decision-making in the near future.

## 1. Introduction

**Figure 1:**
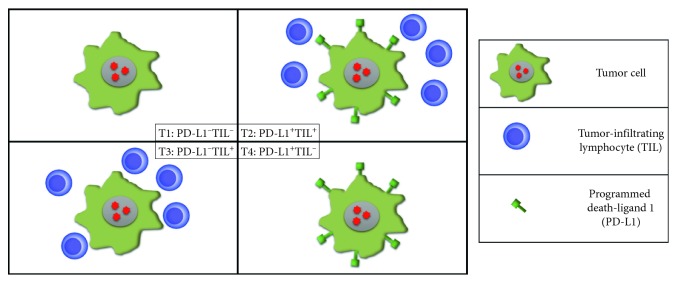
Four tumor subtypes according to the TIME classification based on the expression of PD-L1 in tumor cells and on the presence of TILs.

Immunotherapy is fast becoming the new frontier of oncology, accompanied by the dream of being able to defeat cancer definitively. Although a substantial improvement in survival has been seen since immunotherapy was first used in melanoma, response remains low. The use of different types of immune-checkpoint inhibitors (ICIs), in particular the programmed death-1/programmed death-ligand 1 (PD-1/PD-L1) axis, has led to significantly better results in terms of response and manageability. In recent years, advances have been made in the treatment of urological tumors, especially renal cell cancer (RCC) and urothelial cancer (UC). However, the issue of the identification of nonresponding patients persists. According to the tumor immunity in the microenvironment (TIME) classification [1], tumors can be divided into 4 subgroups based on the presence of inflammatory infiltrate (TIL) and PD-L1 expression: T1 (PD-L1^−^, TIL^−^), T2 (PD-L1^+^, TIL^+^), T3 (PD-L1^−^, TIL^+^), and T4 (PD-L1^+^, TIL^−^) (Figure 1). Although the TIME classification has significant predictive implications, there is an increasing need to find predictive markers of response to ICIs.

## 2. Factors Involved in Primary and Secondary Resistance to ICIs in Solid Tumors

**Figure 2:**
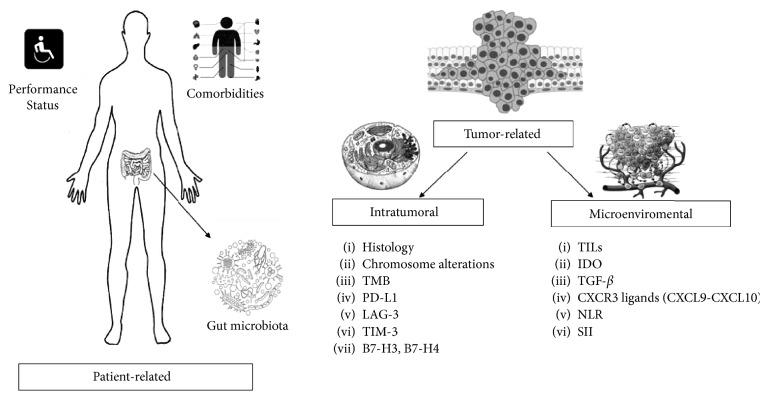
Factors influencing immune response and possibly related to resistance to immunotherapy. TMB: tumor mutational burden; PD-L1: programmed death-ligand 1; LAG-3: lymphocyte-activation gene-3; TIM-3: T-cell immunoglobulin and mucin domain 3; TILs: tumor-infiltrating lymphocytes; IDO: indoleamine-2,3-dioxygenase; TGF-*β*: transforming growth factor-*β*; CXCR: CXC chemokine receptors; CXCL: CXC chemokine receptors ligands; NLR: neutrophil-to-lymphocyte ratio; SII: systemic immune-inflammation index.

Several factors can directly or indirectly influence the immune response and therefore contribute to triggering resistance mechanisms. As shown in Figure 2, these factors can be divided into two categories:

1. Patient-related factors: it is acknowledged that patients in poor clinical conditions have a lower immune response. However, the underlying mechanism for this is still not understood. In fact, Pan et al. reported that an Eastern Cooperative Oncology Group performance score (ECOG PS) of 2 in melanoma patients was associated with worse prognosis when ICIs were used [2]. Conversely, a study carried out on patients with UC treated with atezolizumab showed that response rates (RRs) did not differ among patients with different PS [3]. Recently, several trials conducted on UC demonstrated a shorter overall survival (OS) in patients with ECOG PS > 2 compared with ECOG PS 0 [3–6]. Several comorbidities can also affect the immune response: autoimmune diseases [7, 8], diabetes [9], transplantations [10–12] (including bone marrow transplants), and infections [13]. Another important host-related factor is gut microbiota: several studies have shown that restoration of some bacterial families (Ruminococcaceae [14], *Akkermansia muciniphila* [15], and *Bacteroides fragilis* [16]) is correlated with a longer response in melanoma mice treated with anti-PD1 drugs. Thus, the use of antibiotics or steroids during ICI therapy may affect the outcome of treatment. In particular, 2 recent studies [17, 18] showed that the use of beta-lactams, quinolones, and macrolides during ICIs therapy also led to shorter progression-free survival (PFS) and poorer RR in RCC patients.
2. Tumor-related factors: this category can be divided into 2 subcategories: intratumoral and microenvironmental factors.

### 2.1. Intratumoral Factors

Among tumor-related factors, different histologies and the presence of chromosomal alterations influence the immune response. For example, strongly aneuploid tumors have shown an intrinsic resistance to ICIs [19]. This is due to the poor expression of markers capable of activating the immune response. Conversely, a high expression of mutations, i.e., tumor mutational burden (TMB), especially if mismatch repair genes are involved, correlates with a high RR to ICIs, regardless of histology [20–23]. In UC, a recent study showed a higher RR in patients with alterations in the following genes: ATM, BRCA2, ERCC2, FANCA, MSH6, and POLE [24]. However, unlike solid tumors, elevated TMB has been associated with poor prognosis in hematological cancers, for example, multiple myeloma [25]. The growing interest in TMB has led to the development of studies aimed at testing the efficacy of neoantigens, structured within new molecules, such as chimeric antigen T-cell receptor therapy (CAR-T). Several studies are also underway for patients with RCC [26–28] and prostate tumors (PCa) [29].

PD-L1 expression in tumor tissue is one of the best known mechanisms for neutralizing immune system activity. A higher PD-L1 expression results in a poorer prognosis without the use of ICIs [13]. However, PD-L1 is not always capable of predicting response to ICIs [30, 31]. In fact, although response rates in UC differ significantly on the basis of PD-L1 status, this is not the case for RCC patients [32, 33].

To date, CTLA-4 and PD1/PD-L1 axis are not the only molecules involved in the modulation of the immune response. Other molecules are currently under investigation as potential immune checkpoint for new ICIs, e.g., lymphocyte-activation gene-3 (LAG-3), T-cell immunoglobulin mucin-3 (TIM-3), and B7-H3 and B7-H4/B7x/B7S1.

LAG-3 molecule is located on the cell surface of several immune cells; its ligand is Class II MHC and binds with higher affinity than CD4 [34]. LAG-3 downregulates the immune response of CD4^+^- and CD8^+^-activated cells. In fact, its negative activity has been observed in CD8^+^ tumor-infiltrating lymphocytes (TILs) and in CD4^+^ TRegs [35].

TIM-3 is a regulatory molecule expressed on the surface of innate immune cells; CD8^+^ TILs usually coexpress PD-L1 and TIM-3, causing a strong inhibition of cytokine secretion [36]. To date, TIM-3/PD-L1 coexpression has also been studied in CD8^+^ cells in melanoma patients. In one study, blocking both PD-L1 and TIM-3 led to a restoration of cytokine secretion [37].

B7-H3 and B7-H4 (also known as B7x/B7S1) are 2 members of the B7 super-family expressed not only by immune cells but also by nonlymphoid tissues, including prostate and testis cells [38]. Although B7-H3 was initially characterized as a costimulatory molecule, recent studies have indicated its dual activity. In some cases, it acts as an upregulator of the immune responses and in others, a downregulator [39].

### 2.2. Microenvironmental Factors

Tumor microenvironment plays an important role in silencing the immune response. Usually, the presence of TILs is related to higher PD-L1 expression [40, 41] and to better response to ICI treatment [23]. The KEYNOTE 028 study tested the efficacy of pembrolizumab in 20 different tumors. Results showed that treatment with ICIs was more effective in patients with TILs, independently of tumor histology [42].

On the other hand, the aforementioned TIME classification [1] has emphasized the link between TILs and PD-L1 in determining the response to ICIs. However, its correlation with response in UTs is still under evaluation [43]. The T2 subgroup, for example, is characterized by the presence of TILs and higher PD-L1 expression, stimulated by the TIL-mediated production of interferon-gamma (IFN-*γ*). This subgroup is associated with high RRs when treated with ICIs. Unlike T2, the T3 subgroup expresses TILs but not PD-L1 (probably due to a nonexpression of inducing factors, such as IFN-*γ*). In this context, the use of OX-40 or 4-1BB agonists may convert tumors classified as T3 into T2 [44, 45]. T1 and T4 subgroups differ because of their lack of TILs. Many tumors have this characteristic, which is usually associated with a nonresponse to treatment with ICIs. There are different ways to stimulate the immune response, for example, by using anti-CTLA4 antibodies or CAR-T-cell therapy. However, some negative PD-L1 tumors may respond to an anti-PD-L1 drug. Positivity or negativity of the histological examination may not reflect a common characteristic of the overall tumor. Thus, tumor heterogeneity may be responsible for ICI response in patients with PD-L1-negative biopsy [1]. It is also a unstable characteristic over time; in fact, treatment may select altered tumor cells capable of activating the process of immune escape, blocking the immune system activation, and even transforming positive TIL into negative TIL tumors. This condition has been described in different tumor types, such as lung and breast cancer and RCC [46–48]. In particular, discordance in PD-L1 status between primary and metastatic sites has been observed in 20% of RCC patients [49]. The immune-silencing process is ascribed to several mechanisms: activation of the Wnt–*β*-catenin pathway [50]; loss of PTEN associated with AKT activation [51]; and loss of immunogenicity [52] through several mechanisms (including downregulation of MHC class I molecules and reduced production of immunogenic antigens).

The study of the tumor microenvironment has led to the discovery of other molecules involved in immune-silencing mechanisms. For example, indoleamine-2,3-dioxygenase (IDO) is a molecule produced in TILs capable of stimulating the immune infiltrate, reducing the concentration of tryptophan which is necessary for the activation of cytotoxic T cells, and permitting their transformation into regulatory T cells (TRegs). This promotes an immunosuppressive microenvironment near the tumor. Consequently, IDO is a promising biomarker, and high concentrations are associated with worse prognosis. However, IDO as a target for new drug development has been disappointing, and the use of IDO inhibitors has not shown any advantages over ICI treatment [53]. In addition to IDO, there is a high expression of other molecules in tumor microenvironment, including TGF-*β* secreted by fibroblasts [54], and various other cytokines involved in immune-silencing mechanisms. Among these molecules, CXCL9 and CXCL10, two CXCR3 ligands, have shown to be correlated with the TIL-positive TIME subgroups, whereas TIL-negative subgroups lack these chemokines [55, 56].

Furthermore, several studies have evaluated the prognostic/predictive role of some parameters, such as the neutrophil-to-lymphocyte ratio (NLR) and the systemic immune-inflammation index (SII). NLR is the most widely tested prognostic index and correlates with prognosis in different tumor types [57]. Similarly, SII, combining neutrophils, lymphocytes, and platelet count in a single parameter, demonstrates a significant correlation with prognosis in different cancers [58–60]. Among UTs, SII and NLR have shown a prognostic and predictive role of response to conventional treatment in several retrospective trials [61–63]. In particular, Lalani et al. recently demonstrated that an early reduction in NLR (at 6 weeks) was associated with a significantly improved outcome in mRCC patients after ICI treatment [64]. Moreover, Raccioppi et al. found that preoperatory NLR value was a predictor of response to BCG therapy in non-muscle-invasive bladder cancer [65].

## 3. Potential Prognostic and Predictive Biomarkers in UCs Treated with ICIs

### 3.1. PD-L1 and TILs

PD-L1 is the most widely studied (potential) biomarker in immunotherapy, and several studies have investigated its predictive value in UCs.  Table 1 lists the clinical trials that evaluated PD-L1 expression by immunohistochemistry (IHC) or the IHC-based combined positive score (CPS) to develop a reproducible PD-L1 scoring method that can be used to identify patients most likely to respond to therapy. CPS is obtained as follows: CPS = 100 × PD-L1 stained cells (tumor cells, lymphocytes, macrophages)/total viable tumor cells. In RCC, PD-L1 is not a useful predictor of response to ICI treatment. Both PD-L1-negative and PD-L1-positive tumors respond to immunotherapy, despite higher rates of RR and PFS in patients with PD-L1 expression. In fact, in the metastatic RCC population of the CheckMate 214 trial, the combination of nivolumab plus ipilimumab obtained an objective RR of 37% in patients with PD-L1 expression <1%, compared to 58% of those with PD-L1 expression >1% [31]. In the IMmotion 151 trial, patients with PD-L1 ≥ 1% showed longer PFS when treated with bevacizumab plus atezolizumab [66]. Conversely, the combination of axitinib with pembrolizumab (KEYNOTE 423 trial) or axitinib with avelumab (Javelin Renal 101) did not produce different efficacy results on the basis of different PD-L1 statuses [67, 68]. Similarly, Motzer et al. observed that the use of nivolumab after treatment with anti-VEGFR inhibitors improved OS independently of PD-L1 status [69]. Unlike RCC, PD-L1 has been recognized as a predictive biomarker in UCs. In metastatic/locally advanced UC, atezolizumab and pembrolizumab demonstrated antitumor activity and acceptable tolerability in the first-line treatment of cisplatin-ineligible patients [3, 5]. Based on these results, the Food and Drug Administration (FDA) approved atezolizumab and pembrolizumab in this subgroup. However, the FDA updated the prescribing information for first-line pembrolizumab and atezolizumab in cisplatin-ineligible patients, making it compulsory to use an approved PD-L1 diagnostic test (Dako PDL-1 ICH 22C_3_ PharmDx Assay® and Ventana PDL-1 Assay®) to select patients. Therefore, FDA indications were modified as follows: cisplatin-unfit patients are eligible for pembrolizumab and atezolizumab if the tumor expresses PD-L1 (CPS ≥ 10 for pembrolizumab and PD-L1 ≥ 5% for atezolizumab) [70]. In patients not eligible for any platinum, pembrolizumab and atezolizumab can be administered in first-line regardless of tumor PD-L1 expression. In postplatinum UC patients, several trials have demonstrated ICI efficacy [71–75], with ICI-treated PD-L1-positive TIL-positive UCs showing higher RRs. In the IMvigor 210 trial, the use of atezolizumab obtained an overall response rate (ORR) of 16%, which was higher (28%) in patients with ≥5% PD-L1 expression [71]. In CheckMate 275, patients with tumor cluster III proved most likely to obtain a better response to nivolumab (30%) [73]. Similar results were obtained in 2 other studies. In the JAVELIN trial, avelumab demonstrated an ORR of 17% in all patients and 50% in those showing PD-L1 expression [75]. In a phase 1/2 trial, durvalumab obtained an ORR of 31% in the overall population, 46% in patients with PD-L1 expression, and 0% in those without PD-L1 expression [76]. Based on these results, the FDA approved pembrolizumab as the preferred drug, with atezolizumab, nivolumab, and durvalumab as alternative preferred agents, regardless of PD-L1 expression. The European Medicines Agency (EMA) recently approved pembrolizumab for the treatment of metastatic/unresectable UCs in relapsed patients after first-line platinum-based therapy and also in nonpretreated cisplatin-unfit patients with CPS>10. The EMA has also approved atezolizumab for the first- and second-line treatment of UC and nivolumab for use in a second-line setting. Although the cancer vaccine, sipuleucel-T, has shown activity in prolonging OS in PCa, none of the new ICIs have been approved. This is due to limited antitumor immune infiltrates and poor PD-L1 expression in this tumor type [77, 78]. In germ-cell tumors, PD-L1 expression has been observed in 73% and 64% of patients with seminoma and nonseminoma types, respectively [79] and correlates with outcome. Low levels of PD-L1 are associated with better PFS [80]. Despite the prognostic value of PD-L1 expression, pembrolizumab has not shown activity as a single agent in the treatment of refractory germ-cell tumors [81]. Therefore, PD-L1 is the only recognized biomarker in patients with UC, but its prognostic and predictive role is still open to debate in nonurothelial urological tumors. A recent study of 160 UC patients showed that although PD-L1 positivity ≥5% in tumor cells was not predictive of OS, it was predictive if expressed in TIL cells [82]. Mariathasan et al., after evaluating data from the IMvigor 210 phase 2 trials, reported that differences in PD-L1 also existed between tumor cells and inflammatory cells in TILs [54]. Hence, the debate about the different value of PD-L1 expression in tumor and nontumor cells (TILs) is still open.

### 3.2. Prognostic and Predictive Role of TIM-3, B7-H3, and B7-H4

Tumor-associated macrophages induce a more immunosuppressive phenotype, leading to an enhanced expression of TIM-3 and PD-1 on CD4^+^ and CD8^+^ T cells. The concentration of TIM-3 and PD-1-positive CD4^+^ and CD8^+^ T cells is higher in TILs than in peripheral blood in RCC patients [83]. Recently, Granier et al. demonstrated that PD-1^+^Tim-3^+^CD8^+^ T cells could not be enhanced *in vitro* by a strong stimulus, suggesting that these cells cannot be reactivated after PD-1-PD-L1 blockade [84]. In PCa patients, malignant cells show higher TIM-3 expression than benign cells, expression correlating with TNM staging system, grading, and PFS [85]. Piao et al. demonstrated that Tim-3 expression in both CD4+ and CD8+ T cells closely correlated with advanced disease and poor prognosis in PCa patients [86]. Other studies have evaluated the prognostic role B7-H3 and B7-H4 in UTs. In both RCC and PCa, the overexpression of B7-H3 and B7-H4 was correlated with poor prognosis and a higher risk of recurrent and metastatic disease [87, 88]. Moreover, in RCC, B7-H3 and B7-H4 were expressed by both immune and endothelial cells: among 743 RCC patients, B7-H3-positive TILs were observed in 17% of tumor samples and in 95% of tumor vasculature [89]. Another study reported a B7-H4 positive expression in tumor vasculature of 211 RCC patients [90, 91]. In UCs, B7-H3 is overexpressed in all tumor stages and its expression can be stimulated by Bacillus Calmette–Guérin-based therapy [92].

### 3.3. Prognostic Role of NLR and SII

In the last few years, the prognostic role of NLR and SII has been evaluated in urological and nonurological cancers. Although several studies have demonstrated a correlation between NLR and prognosis and NLR and treatment response, its prognostic role remains uncertain [93, 94]. In UC and RCC, NLR is significantly associated with prognosis [95–97]. As seen in breast cancer [98], lymphopenia is also associated with poor prognosis in patients with RCC [99]. In a study on an elderly mRCC population treated with first-line sunitinib, lymphopenia proved to be a negative prognostic factor [100]. Thrombocytosis has also been identified as a negative prognostic factor in RCC patients [101]. A recently published study evaluated the role of SII in RCC patients treated with the PD-1 inhibitor nivolumab and enrolled in an Italian Expanded Access Program. The authors demonstrated that normal body mass index combined with higher SII tripled the risk of death, suggesting that SII is a critical prognostic factor for OS in pretreated RCC patients during treatment with nivolumab [102]. A recent article confirmed the prognostic role of SII (and its variations during therapy) in mRCC patients treated with sunitinib [103]. Recently, a study evaluated the combination of SII and the monocyte/lymphocyte ratio (MLR) as new prognostic factor in upper-tract UC. The authors demonstrated that SII was significantly associated with PFS and OS, whereas MLR significantly correlated with OS but not with PFS. Both SII and MLR correlate with an enhanced risk of disseminated disease [104]. In PCa, Fan et al. reported that SII has a negative independent prognostic role in terms of OS in patients treated with both abiraterone and docetaxel, independently of the treatment sequence [105].

### 3.4. Predictive Role of IFN-*γ* and Other Cytokines

A 25-gene IFN-*γ* signature was evaluated in patients with metastatic UC enrolled in the phase II trial CheckMate 275, a trial focusing nivolumab used as a single agent. The analysis demonstrated that a higher IFN-*γ* signature was expressed in the basal-1 subgroup, corresponding to cluster III of the TCGA classification. The patients in this group were more likely to respond to ICIs [72, 73]. Recently, IFN-*γ*-induced cytokines (CXCL9 and CXCL10) were also shown to be positive predictors of response to atezolizumab in the IMvigor trial [71].

### 3.5. Prognostic and Predictive Role of TMB and Genetic Instability

In PCa, 2 large phase III trials on unselected patients reported the failure of anti-CTLA4 (ipilimumab) [106, 107]. Initial clinical data had shown that 5%–12% of patients with metastatic PCa may benefit from ICIs [108, 109], probably due to the low mutational loads of PCa, which is correlated with low neoantigen burden [110]. The mismatch repair (MMR) gene is a DNA single-strand repair mechanism. Mismatch repair-deficient (dMMR) cancers are characterized by microsatellite instability and hypermutator phenotype, both associated with chemotherapy resistance but immunotherapy sensitivity [111]. In a study by Iyer et al., dMMR or high MSI (MSI-H) were found in 3% of 424 UC patients [112], both subgroups showing a higher response to ICIs [112]. A recently published phase II trial including patients with cholangiocarcinoma, colorectal, endometrial, gastric, and small bowel cancer demonstrated that dMMR predicted clinical benefit from pembrolizumab [20]. In PCa, the prevalence of dMMR varies between 12% and 22% in different studies, probably because of the different assays used to detect the genomic aberrations [113, 114]. Recent evidence that dMMR cancers may benefit from pembrolizumab [20] has led to FDA approval of pembrolizumab for the treatment of metastatic/unresectable solid tumors with dMMR or MSI-H in patients who progress on prior treatment. Initially, this indication included several cancer types but not PCa. After the results from the KEYNOTE-028-phase 1b trial were published [109], the FDA expanded the previous indication to include patients with pretreated metastatic PCa with MSI-H or dMMR deficiency [115]. However, dMMR cancers do not always respond to immunotherapy, and not all cancers responding to ICIs are dMMR [20, 21, 116]. In fact, a recent study showed that dMMR tumors constitute a subtype with decreased survival time but that only a proportion has a high mutation load and show PD-L1 IHC staining. Thus, dMMR tumors represent a heterogeneous group and may require further subclassification to understand their clinical behaviour and response to ICIs [117]. However, NCCN guidelines still recommend DNA-repair gene mutation testing for all patients with high-risk regional or metastatic PCa [115].

## 4. Conclusions

In UCs, several ICIs have been approved in metastatic disease and several studies are ongoing in a nonmetastatic setting. To date, 2 biomarkers have been recognized in clinical practice: PD-L1 and dMMR. The FDA and EMA permit the use of pembrolizumab and atezolizumab in UC cisplatin-ineligible patients expressing PD-L1 and undergoing first-line treatment for metastatic disease. The presence of dMMR or MSI-H also represents a predictive factor of response to ICIs in PCa and has led to FDA approval of pembrolizumab in this subgroup. Notwithstanding, several unanswered questions remain: Why do some tumors express TILs and some do not? Why do some tumors not express PD-L1? What regulates immune escape mechanisms? The role of PD-1 and PD-L1 expression as a predictive biomarker is still unclear, the use of different methods and cutoff points in trials complicating its validation. As suggested by Mariathasan et al., another difference may derive from different PD-L1 expressions in both tumor cells and immune cells [54]. Moreover, patients with low or negative PD-L1 expression respond to ICIs. Consequently, more suitable biomarkers must be sought. In the near future, it is hoped that the biological characterization of tumors will be able to drive clinical decision-making, leading to more personalized treatment. In UCs, new classification systems such as TCGA will add further valuable information, allowing for better patient selection. Furthermore, classification of biomarker expression into the three immunological phenotypes “immune inflamed,” “immune excluded,” and “immune desert” could improve our knowledge of distinct immunological pathways, enabling a more effective use of ICIs such as mono- or combination therapies [118].

In the past, nanoparticle-based drugs have been hypothesized for the treatment of cancer. These drug nanocarriers can improve the therapeutic efficacy of a drug by penetrating deep into tissue and overcoming the physical barriers linked to drug release [119]. In this scenario, the identification of new cancer-specific biomarkers could lead to the development of new nanocarrier drugs directed against cancer-specific driver biomarkers. In the near future, the identification of new biomarkers capable of predicting outcome and of acting as molecular targets for cancer treatment will be possible, thanks to a greater understanding of the intrinsic mechanisms that regulate immune system activity. Meanwhile, the search for new and reliable predictive biomarkers will proceed in 3 main directions: humoral (cytokines), immunohistochemical (new or unexplored checkpoints), and genomic (mutations, genetic instability).

## Figures and Tables

**Table 1:** Potential predictive biomarkers in urological tumors treated with ICIs.

Histology	Biomarker	Trial/author	Drugs	Setting	Study results
Urothelial	PD-L1 (CPS)	KEYNOTE 052 (phase 2)	Pembrolizumab	1-line CDDP ineligible	24% ORR, highest ORR in patients with CPS ≥ 10%
	PD-L1 (CPS)	KEYNOTE 045 (phase 3)	Pembrolizumab vs CHT	Second line after platinum-based CHT	Higher ORR in pembrolizumab group than CHT, regardless of tumor PD-L1 expression
	PD-L1 (IHC)	NCT02108652 (phase 2)	Atezolizumab	≥2-line after platinum-based CHT (cohort 2)	ORR: 26% (PD-L1 ≥ 5%) vs 15% (all patients) OS: 11.4 (PD-L1 ≥ 5%) vs 7.9 (all patients) months
	PD-L1 (IHC)	NCT02108652 (phase 2)	Atezolizumab	First-line CDDP ineligible	No significant enrichment of response and OS by PD-L1 expression
	PD-L1 (IHC)	NCT01772004 (phase 1b)	Avelumab	≥2-line treatment after platinum-based CHT	Patients with higher PD-L1 ≥ 5% showed higher response rates and longer PFS and OS
	PD-L1 (IHC)	CheckMate 275 (phase 2)	Nivolumab	≥2-line treatment after platinum-based CHT	ORR: 28.4% (PD-L1 ≥ 5%) vs 23.8% (PD-L1 ≥ 1%) vs 16.1 (PD-L1 < 1%); OS: 11.3 (PD-L1 ≥ 1%) vs 5.9 (PD-L1 < 1%) months
	CXCL9, CXCL10 cytokines	CheckMate 275 (phase 2)	Nivolumab	≥2-line treatment after platinum-based CHT	Positive predictors of response to nivolumab
	CXCL9, CXCL10 cytokines PD-L1 rabbit SP142 (Ventana)	IMvigor 210 (phase 2)	Atezolizumab	≥2-line after platinum-based CHT (cohort 2)	Positive predictors of response to atezolizumab; PD-L1 expression on IC (>5% of cells) was significantly associated with response. In contrast, PD-L1 expression in tumor cells was not associated with response
	PD-L1 (IHC)	NCT01693562 (phase 2)	Durvalumab	≥2-line treatment after platinum-based CHT	No differences in PFS and ORR between high and low/negative PD-L1 patients
	dMMR or MSI-H	G. Iyer et al., J Clin Oncol 2017	ICIs	Metastatic setting	dMMR caused a high mutation load and was associated to durable responses to ICIs
				

Kidney	PD-L1 rabbit 28-8 (Dako)	CheckMate 214 (phase 3)	Nivolumab ipilimumab vs sunitinib	First line	Greater benefit in ORR, PFS, and OS for patients with PD-L1 ≥ 1% treated with nivolumab and ipilimumab
	PD-L1 (IHC)	Javelin renal 101	Avelumab plus axitinib vs sunitinib	First line	Greater benefit in ORR and PFS in patients with treated with avelumab plus axitinib, independently from PD-L1
	PD-L1 (IHC)	KEYNOTE 423 (phase 3)	Pembrolizumab plus axitinib vs sunitinib	First line	Greater benefit in ORR, OS, and PFS in patients with treated with pembrolizumab plus axitinib, independently of PD-L1
	PD-L1 (IHC) rabbit SP142 (Ventana)	IMmotion 151 (phase 3)	Bevacizumab/atezolizumab vs sunitinib	1-line	PFS in PD-L1 ≥ 1% patients: 11.2 mo (with atezolizumab plus bevacizumab) vs 7.7 mo (with sutent), HR 0.74, *P* = 0.0217
	PD-L1 (IHC) rabbit 28-8 (Dako)	CheckMate 025 (phase 3)	Nivolumab vs everolimus	≥2-line treatment after anti-VEGFR therapy	No differences in OS on the basis of PD-L1 status
	SII rabbit 28-8 (Dako)	De Giorgi et al., Clin Cancer Research 2019	Retrospective analysis of EAP of nivolumab	≥2-line treatment after anti-VEGFR therapy	Normal body mass index combined with higher SII tripled the risk of death
				

Prostate	dMMR	Le DT et al., Science 2017	Pembrolizumab	Advanced dMMR cancers	ORR: 53% of patients and complete responses were achieved in 21% of patients

PD-L1 = programmed death-ligand 1; CPS = combined positive score; ICIs = immune-checkpoint inhibitors; ICH = immunohistochemistry; SII = systemic inflammation index; dMMR = mismatch repair genes deficiency; MSI-H = higher microsatellite instability; CHT = chemotherapy; EAP = expanded access program; ORR = overall response rate; PFS = progression-free survival; OS = overall survival.
